# Association of interictal epileptiform discharges and serum concentration of levetiracetam and lamotrigine

**DOI:** 10.3389/fneur.2024.1524637

**Published:** 2025-01-24

**Authors:** Johannes D. Lang, Alexander Willno, Caroline Reindl, Tamara Welte, Jenny Stritzelberger, Stephanie Gollwitzer, Katrin Walther, Hajo Hamer

**Affiliations:** Epilepsy Centre, Department of Neurology, University Hospital Erlangen, Erlangen, Germany

**Keywords:** spikes, interictal epileptiform discharges, anti-seizure medication, anticonvulsants, antiepileptic drugs

## Abstract

**Background:**

Interictal epileptiform discharges (IEDs) are an electrographic biomarker of epilepsy. Despite their crucial role in diagnosing epilepsy, heterogeneous findings exist on the mechanisms underlying their occurrence and the effects of anti-seizure medications (ASMs) on IEDs.

**Methods:**

We conducted a study to investigate the association between IED frequency and the serum concentration of two commonly used ASMs, levetiracetam (LEV) and lamotrigine (LTG). We included 56 patients undergoing a continuous video EEG monitoring in our center with tapering of ASM. IED frequency was analyzed using automated and semiautomated methods and serum samples were collected sequentially throughout the stay.

**Results:**

The cohort consisted of 41 patients (23 female, 18 male), between 19 and 64 years (mean 37.42 years), most of which were diagnosed with focal epilepsy (93%). IED frequency increased after ASM reduction revealing a negative correlation similarly with LEV and LTG serum concentrations (*p* = 0.0057 and *p* = 0.0426, respectively).

**Discussion:**

Notably, we observed a significant increase in IED frequency following dose reduction or discontinuation of both medications. This effect was reversed after ASM were re-dosed. This may indicate the suppressive properties of LEV and LTG against epileptic seizures. Furthermore, our study highlights the importance of ASM discontinuation, which may be required for capturing IEDs during diagnostic continuous EEG monitoring, and not be fully explained by circadian or ultradian rhythms alone.

**Conclusion:**

Our findings contribute to the understanding of ASM effects on IED frequency dynamics and suggest seizure suppressive properties of LEV and LTG.

## Introduction

The recurrence of unprovoked epileptic seizures is the decisive factor in the definition of epilepsy (1). However, due to their relatively unpredictable occurrence, recording them in a routine electroencephalogram (EEG) is not trivial and often reserved for a long-term EEG or a stay at an EEG monitoring unit. Interictal epileptiform discharges (IED), on the other hand, are more common and therefore easier to detect transients of electrical cortical activity of different duration and configuration. Their strong association with epilepsy and their occurrence in the interictal interval, i.e., between two successive seizures, have made IEDs a biomarker of epileptic activity and a powerful screening instrument for identifying and classifying epilepsies (2). The brief variants, the so-called “spikes,” are defined as transients with a generally negative main component, a characteristic “sharp peak at conventional paper speed with a duration of 20 to less than 70 ms, and a variable amplitude of >50 μV,” which if followed by a slower wave of the same polarity, are called “spike–wave complexes.” Because of the arbitrary and mostly descriptive distinction between spikes and transients of similar characteristics with a slower time course up to 200 ms, the so-called “sharp waves,” these transients have been grouped together as IEDs (3, 4). Beyond their diagnostic and localizing value, it remains unclear what their exact role is within epilepsies, epileptic seizures, and epileptogenesis, and more fundamental, if their presence represents an excitatory or inhibitory activity within an epileptic network (5).

The significance of their frequency remains unclear. In addition to diurnal fluctuations, conflicting findings on IED frequency in the pre- and postictal interval have been reported (5, 6). While single IED and short sequences of epileptic activity can occur without any recognizable clinical symptoms, short series of generalized spike-and-wave complexes can be associated with impaired awareness and reaction as in absence epilepsy. Furthermore, IED are not exclusively an electrographic phenomenon, but are associated with impaired cerebral perfusion, metabolic changes and can affect cognitive performance (4).

Typically, IEDs are analyzed in terms of their shape, distribution, and frequency. While the first two usually require the averaging of many potentials of similar shape and distribution, the latter compare, e.g., their relative incidence in different states of vigilance, i.e., wakefulness and sleep, in pre- and postictal episodes, and under different drug conditions. Concerning their association with the efficacy of anti-seizure medication, conflicting findings have been reported with both, an increase and decrease of IED frequency under medication (5). Therefore, we designed this study to investigate the association of IED frequency in continuous EEG and the serum concentration of levetiracetam (LEV) and lamotrigine (LTG), two widely used newer agents of anti-seizure medication (ASM) with sequential serum samples over the course of several days.

## Methods

We included 56 patients who were referred to our center for a one-week video EEG monitoring between 2018 and 2020. All patients received a continuous EEG with scalp electrodes according to the 10–20 system (7) for either diagnostic reasons or for a presurgical workup. We included people with confirmed epilepsy if they were at least 18 years old, took either ASM and were admitted to our monitoring unit with the intention of recording epileptic seizures and/or IED for diagnostic purposes, sometimes by reducing or discontinuing medication. Exclusion criteria encompassed pregnant or breastfeeding women, minors and people under legal guardianship or limited legal capacity.

### Sequential blood sampling

After patients gave informed consent, sequential serum samples were drawn every other day, unless dose changes were made, in which case blood was taken daily for 3 days. Dose changes were made solely based on clinical considerations, e.g., to induce epileptic seizures for diagnostic purposes or improve their suppression. Serum concentrations were measured in our clinical laboratory using mass spectrometry.

### IED frequency

After the patients have been discharged, the frequency of IED was determined using Brain Electrical Source Analysis (BESA®) software (BESA GmbH, Gräfelfing, Germany) with two approaches: (1) using an automated IED detection and clustering algorithm in BESA® Epilepsy (version 2), and (2) using a semi-automated template search in BESA® Research (version 6). The automated evaluation was carried out for all recorded EEG, on blocks of 24 h starting at 8 AM, while the semi-automated frequency evaluation was conducted on blocks of two hours every night and day, with the latter containing a standardized awake EEG routine including alpha blocking with visual stimuli (eye opening) and hyperventilation. Since IED frequency differs significantly between individual patients, we calculated the relative frequency based on a baseline, which was established as the mean IED frequency before dose alterations or discontinuation for dose reduction or drug withdrawal, respectively.

### Statistics

All statistics were calculated using JMP® 16.0.0 software. Normal distribution was tested using Shapiro–Wilk W test for absolute and relative measures of IED frequencies and ASM serum concentration for LEV and LTG, which indicated that all variables were not normally distributed (Supplementary Table S1). Consequently, throughout the analysis, we used non-parametric tests, e.g., Wilcoxon rank sum test (group comparison) and Spearman’s rho (correlation).

## Results

### Patient cohort and clinical characteristics

Of 56 patients, 41 complete data sets were o5tained for further analyses. We excluded 15 patients after sampling, due to insufficient number of serum samples (n = 8) or dropped out during the sampling period due to clinical reasons (early discharge and diagnosis other than epilepsy, n = 5), and two patients at own will.

Our final cohort consisted of 41 patients (23 female, 18 male), between 19 and 64 years (mean 37.42 years). An overview of their clinical characteristics is given in Table 1. Most patients were diagnosed with focal epilepsy, only 3 patients suffered from generalized epilepsy. Of these, 19 patients took LEV and 22 were on LTG, and five patients took both ASM (LEV and LTG). Age at (epilepsy) onset ranged from 3 to 55 years. One patient was diagnosed with psychogenic non-epileptic seizures (PNES) in addition to focal epilepsy.

**Table 1:** Cohort and clinical characteristics.

Parameters		Total	LEV	LTG
*N*		41	19	22
Age	(range) [years]	37.42 (19–64)	40.53 (22–64)	36.64 (19–64)
Age at onset	(range) [years]	20.44 (3–55)	22.74 (7–55)	20.14 (3–55)
Gender	Male/female	18/23	9/10	9/13
Epilepsy type	Focal Epilepsy	38	18	20
	Generalized Epilepsy	3	1	2
Hemisphere	Right/left/multifocal		9/8/1	9/10/1
Lobe	Temporal		10	10
	Frontal		5	7
	Parietal		3	1
	Occipital		1	1
	Multifocal		1	1
	Unclear			1

Total cohort, patients under LEV, Levetiracetam, and LTG, Lamotrigine. Age (at inclusion) and age at (epilepsy) onset as mean and range in years, other information in total numbers.

### IED frequency

Across all patients, IED frequency detected by both methods (using BESA® Epilepsy and Research), did not differ significantly when compared according to seizure frequency as recorded by clinical history on admission (Supplementary Figure S1). Therefore, data from BESA® Epilepsy on 24 h per day were used for further analyses, which reached up to 8,067 IED or a maximum of 165-fold relative increase within 24 h. Median IED frequency was 367 and 275 per day for patients taking LEV and LTG, respectively (Supplementary Figure S2).

### Levetiracetam

**Figure 1:**
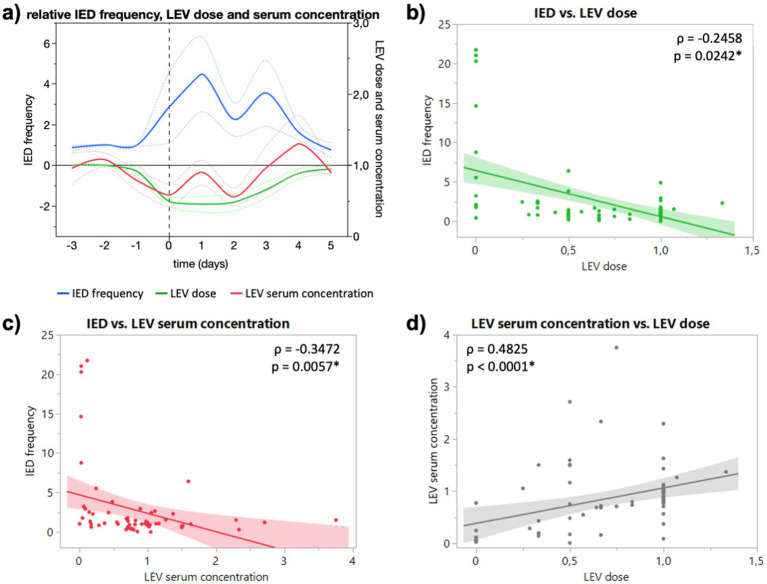
**(A)** Course of relative IED frequency, LEV dose and serum concentration in relation to baseline (days −3 to −1) given as mean (full lines, colors see legend) and standard error of the mean (dotted lines). X axis in days relative to the ASM reduction (0, dashed vertical line). Maximum mean IED frequency 4.5-fold (2.6 to 6.3) one day after dose reduction. **(B)** Correlation of relative IED frequency and LEV dose. **(C)** Correlation of relative IED and LEV serum concentration. **(D)** Correlation of relative LEV dose and LEV serum concentration. Correlation calculated with non-parametric Spearman’s *ρ*.

Under the influence of levetiracetam (LEV), the relative IED frequency showed an increase to 2.8-fold (1.1 to 4.6) on the day of dose reduction and to a maximum of 4.5-fold one day after (2.6 to 6.3). It decreased again when doses were increased (Figure 1A). Relative IED frequency correlated with LEV dose (*p* = 0.0242) and serum concentration (*p* = 0.0057), which in turn correlated with each other (*p* < 0.0001, Figures 1B–D, respectively). In addition, IED frequency correlated significantly with absolute LEV dose (*p* = 0.0156) and serum concentration (*p* = 0.0083; data not shown). For 5 patients under LEV, doses were not altered due to a suspected high number of seizures (>1–2 per week) or recording of seizures within the first 24 h of the monitoring (at baseline), which made dose reduction or discontinuation unnecessary. Interestingly, IED frequency (*p* = 0.2661) and serum concentrations (*p* = 0.3822) did not differ significantly compared to reduction (Supplementary Figure S3).

### Lamotrigine

**Figure 2:**
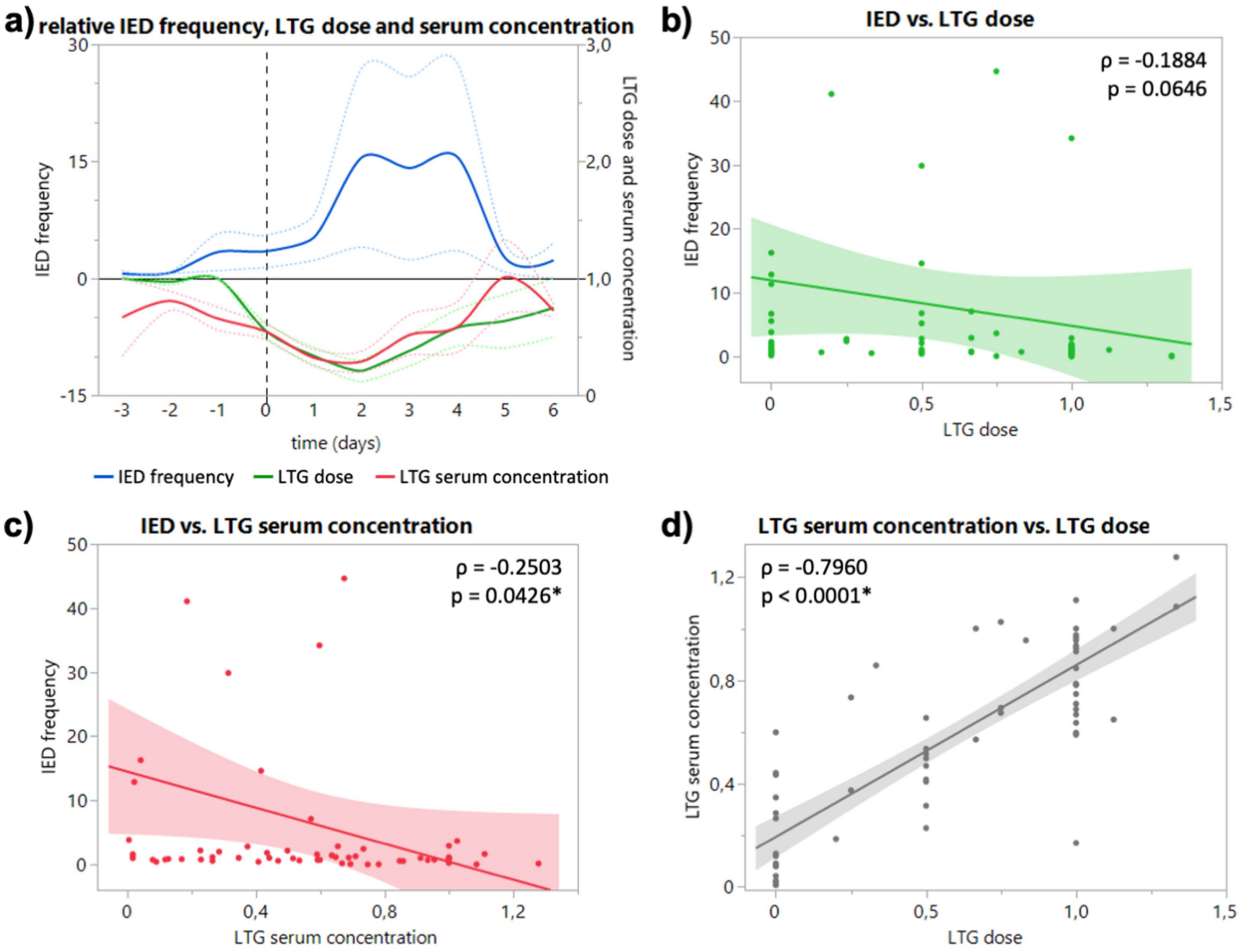
**(A)** Course of relative IED frequency, LTG dose and serum concentration in relation to baseline (days −3 to −1) given as mean (full lines, colors see legend) and standard error of the mean (dotted lines). X axis in days relative to the ASD discontinuation (0, dashed vertical line). Maximum mean IED frequency 15.5-fold 2 and 4 days after dose reduction [15.49 (4.02 to 26.97) and 15.52 (3.55 to 27.49)]. **(B)** Correlation of relative IED frequency and LTG dose. **(C)** Correlation of relative IED and LTG serum concentration. **(D)** Correlation of relative LTG dose and LTG serum concentration. Correlation calculated with non-parametric Spearman’s ρ.

In the EEG of patients who took lamotrigine (LTG), an increase of IED frequency to 3.5-fold (1.4 to 5.6) on the day of dose reduction and to 5.3-fold (2.3 to 8.2) one day after (Figure 2A) can be registered. The maximal mean IED increase was 15.5-fold 2 and 4 days after dose reduction (15.49 and 15.52) (Figure 2A). Relative IED frequency correlated with LTG serum concentration (*p* = 0.0426; Figure 2C), but not with LTG dose (*p* = 0.0646; Figure 2B). Relative IED frequency did not correlate significantly with absolute LTG dose (*p* = 0.1842) and serum concentration (*p* = 0.3005; data not shown). For two patients under LTG, no dose reduction was directed due to recording of seizures within the first 24 h of the monitoring (at baseline).

## Discussion

For both ASM, LEV and LTG, the course of IED frequency on up to 9 days of consecutive recording showed a clear increase to 4.5 and 15.5-fold, respectively between at the day of dose reduction or ASM cessation, and a decrease after re-dosing or reintroduction of the drugs. In fact, IED frequency and serum concentration showed a negative correlation for both substances. This suggests suppressive properties of LEV and LTG against IED. But a review of the literature on this association points to a more complex interaction with evidence for a possible underlying inhibitory activity.

The scientific evidence on IED gathered over the past decades is heterogeneous, with conflicting findings regarding their frequency and association with epileptic seizures and epileptogenesis. Gotman reported that the frequency of IED did not change systematically leading up to epileptic seizures (8). Moreover, he observed an increase in their frequency in the hours and days following epileptic seizures but not after ASM reduction (6). The latter might be explained in parts by the fact, that patients in this similarly sized cohort were not recorded throughout the day, nor following a similar protocol, and did not receive neither LEV nor LTG. In contrast, we obtained the IED frequency from continuous 24-h recordings for all patients and registered an increase of IED frequency following ASM reduction or discontinuation, regardless of the occurrence of epileptic seizures. Our findings are consistent with a more recent study based on continuous recordings, which showed an increase after ASM withdrawal (9). Goncharova et al. confirmed Gotman’s observation of increased frequency with the occurrence of an epileptic seizure in continuous recordings averaging 9 days, which we did not observe in our data (10). However, they reported a decrease in IED frequency with ASM reduction leading up to epileptic seizures. This observation differs from our results and those of De Stefano and may be explained in part by the fact that Goncharova et al. recorded a higher median IED frequency of more than 600 per day. In contrast, we registered a median IED frequency of 367 and 275 per day for patients under LEV and LTG, respectively (Supplementary Figure S2), which may indicate a better seizure control in our cohort or a relevant influence of other dynamics.

Interestingly, ASM serum concentration not dose showed a significant negative correlation with IED frequency for both drugs (Figures 1B,C, 2B,C), although dose correlated significantly with serum concentration for both substances (Figures 1D, 2D). In our study, LTG dose did not correlate significantly with IED frequency, but serum concentration (Figures 2B,C), which could be explained by the slower absorption of LTG (5–6 days to steady-state vs. 2 days LEV). However, it remains unclear if the changes of IED frequency following reduced ASM concentration are of pathophysiological significance. While the suppressive effect of ASM on seizures is well established, the effect of ASM on IED is heterogeneous. Oftentimes the cortical region that generates IED (the so-called irritative zone) does not coincide with the ictal onset zone, and it is subject to an ongoing discussion whether IED originate from an excitatory or inhibitory activity. Although, it is believed that interictal spiking sets a condition that preludes the onset of ictal discharges, they should not be interpreted as pre-ictal phenomena since they are generated by different populations of neurons through different cellular and network mechanisms (5). Furthermore, the strong after-inhibition produced by IED maintains a low level of excitation after an initial neuronal bursting (4), which seems to protect against the occurrence of ictal discharges in a general condition of hyperexcitability determined by the primary epileptogenic dysfunction (5). Combined with the early observations of Gotman, that IED frequency decreases in the run-up to epileptic seizures, the periodicity of IED can be interpreted as sign of focal inhibitory processes opposing the epileptogenic activity (Lebovitz 1979). More interestingly, Goncharova found a higher frequency of IED to be associated with a longer time to the first seizure, which suggests that IED are a marker of inhibition, rather than excitation (Goncharova 2016). In this sense, our finding of an increased IED frequency after reduced or discontinued ASM can be interpreted as an increase of inhibitory network activity (rescue attempt) due to an increased epileptogenic activity. However, this might be different for generalized epilepsies, where LEV reduced not only spike–wave density (spikes per hour, i.e., frequency) and but also duration of generalized IED in refractory IGE (13). Due to the low number of generalized epilepsies, we could not observe a difference for type of epilepsy in our study. Furthermore, the fact that subclinical seizure patterns are frequently observed in continuous invasive EEG blurs the boundaries between interictal and ictal events and might explain the heterogenous observations of the last decades.

Furthermore, it is well established that the IED frequency is also subject to circadian fluctuations with a robust and uniform increase during sleep (14). More precisely, IED frequency depends on the degree of wakefulness or rather sleep stages. In a meta analysis Ng and Pavlova showed that relative to REM sleep (1.0), the IED rate differed significantly between wakefulness (1.11) and NREM, with an increase of 75 percent in N1, 69 percent in N2 and 2.46 times higher rates in N3 (11). In addition to circadian changes, analyzing ultralong recording durations of up to 10 years, revealed that IED frequencies fluctuate with ultradian (12 h) but especially with even slower multidian (10 and 26d) periods (12). However, the changes we observed in our study following dose reductions were of a different order of magnitude (mean increase for LEV ~4 and LTG 15 times higher) and consistent over several days of dose reduction or drug discontinuation (Figures 1A, 2A) and are therefore not sufficiently explained by fluctuations alone. Thus, our study highlights the importance of continuous EEG monitoring in capturing fluctuations in IED frequency, which may not be fully explained by circadian or ultradian rhythms alone. These insights shed light on the complex interplay between ASM treatment, IED dynamics, and epileptic activity, and potentially paving the way for further research into more personalized epilepsy management strategies.

### Limitations

We did not distinguish IED according to spike shape and wave characteristics (spike, sharp wave, spike–wave, etc.) which could add to a more comprehensive picture concerning the effect of ASM, since spikes and spike-waves could have a different role in the presence of an epileptic dysfunction.

We did not specifically investigate the time to seizures and potentially existing dynamics of IED frequencies.

The results ask for a more comprehensive study on a larger cohort with scalp and invasive EEG combined to analyze the effect of an altered ASM serum concentration on different patterns of each neuronal cell type (i.e., pyramidal cells and interneurons) surrounding different groups of IED before and after seizures.

## Conclusion

In the light of other valuable research our findings contribute to the understanding of ASM effects on IED frequency dynamics. The negative correlation suggests suppressive properties of both medications. However, considering their well-documented suppressive effect against epileptic seizures, and the indications of an inhibitory property of IED, the reduction of IED frequency following ASM administration may reflect their seizure-suppressive effect in epilepsy, which would render an inhibitory network activity less important, rather than a suppressive effect against IED themselves.

## Data Availability

The datasets presented in this article are not readily available because while the participants declared their consent to data analysis and publication, they did not consent to any type of sharing data or making it available to third parties. Requests to access the datasets should be directed to johannes.lang@uk-erlangen.de.

## References

[1] Fisher RS Acevedo C Arzimanoglou A Bogacz A Cross JH Elger CE. ILAE official report: a practical clinical definition of epilepsy. Epilepsia. (2014) 55:475–82. doi: 10.1111/epi.12550, PMID: 24730690

[2] Gallotto S Seeck M. EEG biomarker candidates for the identification of epilepsy. Clin Neurophysiol Practice. (2023) 8:32–41. doi: 10.1016/j.cnp.2022.11.004, PMID: 36632368 PMC9826889

[3] Chatrian GE Bergamini L Dondey M Klass DW Lennox-Buchthal M Petersen I. A glossary of terms Most commonly used by clinical Electroencephalographers: G. E. Chatrian (chairman), L. Bergamini, M. Dondey, D. W. Klass, M. Lennox-Buchthal and I. Petersén. Electroencephalogr Clin Neurophysiol. (1974) 37:538–48. doi: 10.1016/0013-4694(74)90099-6, PMID: 4138729

[4] Rodin E Constantino T Rampp S Wong PK. Spikes and epilepsy. Clin EEG Neurosci. (2009) 40:288–99. doi: 10.1177/155005940904000411, PMID: 19780350

[5] De Curtis M Avanzini G. Interictal spikes in focal Epileptogenesis. Prog Neurobiol. (2001) 63:541–67. doi: 10.1016/S0301-0082(00)00026-5, PMID: 11164621

[6] Gotman J Marciani MG. Electroencephalographic spiking activity, drug levels, and seizure Occurence in epileptic patients. Ann Neurol. (1985) 17:597–603. doi: 10.1002/ana.410170612, PMID: 3927818

[7] Klem GH Lüders HO Jasper HH Elger C. The ten-twenty electrode system of the international federation. The International Federation of Clinical Neurophysiology. Electroencephalogr Clin Neurophysiol Suppl. (1999) 52:3–6. PMID: 10590970

[8] Gotman J Koffler DJ. Interictal spiking increases after seizures but does not after decrease in medication. Electroencephalogr Clin Neurophysiol. (1989) 72:7–15. doi: 10.1016/0013-4694(89)90026-6, PMID: 2464478

[9] De Stefano P Ménétré E Vulliémoz S Van Mierlo P Seeck M. Changes of interictal epileptiform discharges during medication withdrawal and seizures: a scalp EEG marker of epileptogenicity. Clin Neurophysiol Pract. (2022) 7:279–84. doi: 10.1016/j.cnp.2022.09.004, PMID: 36312513 PMC9615133

[10] Goncharova II Alkawadri R Gaspard N Duckrow RB Spencer DD Hirsch LJ. The relationship between seizures, Interictal spikes and antiepileptic drugs. Clin Neurophysiol. (2016) 127:3180–6. doi: 10.1016/J.CLINPH.2016.05.014, PMID: 27292227

[11] Ng M Pavlova M. Why are seizures rare in rapid eye movement sleep? Review of the frequency of seizures in different sleep stages. Epilepsy Res Treat. (2013) 2013:1–10. doi: 10.1155/2013/932790, PMID: 23853720 PMC3703322

[12] Baud MO Kleen JK Mirro EA Andrechak JC King-Stephens D Chang EF. Multi-day rhythms modulate seizure risk in epilepsy. Nat Commun. (2018) 9:88. doi: 10.1038/s41467-017-02577-y, PMID: 29311566 PMC5758806

[13] Rocamora R Wagner K Schulze-Bonhage A. Levetiracetam reduces frequency and duration of epileptic activity in patients with refractory primary generalized epilepsy. Seizure. (2006) 15:428–33. doi: 10.1016/j.seizure.2006.05.012, PMID: 16837220

[14] Spencer DC Sun FT Brown SN Jobst BC Fountain NB Wong VSS. Circadian and ultradian patterns of epileptiform discharges differ by seizure-onset location during long-term ambulatory intracranial monitoring. Epilepsia. (2016) 57:1495–502. 27396544 10.1111/epi.13455

