# Supplementary material for: Association of interictal epileptiform discharges and serum concentration of levetiracetam and lamotrigine
Source: Front Neurol. 2025 Jan 24;15:1524637. doi: 10.3389/fneur.2024.1524637 (PMC11802410; doi:10.3389/fneur.2024.1524637)
Supplement: Supplementary file 1 [file Data_Sheet_1.docx]

Association of interictal epileptiform discharges and serum concentration of levetiracetam and lamotrigine

Johannes D. Lang^1*^, Alexander Willno^1^, Caroline Reindl^1^, Tamara Welte^1^, Jenny Stritzelberger^1^, Stephanie Gollwitzer^1^, Katrin Walther^1^, Hajo Hamer^1^

^1^Epilepsy Centre, Department of Neurology, University Hospital Erlangen, Erlangen, Germany

***Correspondence:**Johannes Lang, MD
Epilepsy Centre
Department of Neurology
University Hospital Erlangen
Schwabachanlage 6
91054 Erlangen, Germany
Johannes.lang@uk-erlangen.de

# Supplementary Data

none

# Supplementary Figures and Tables

## Supplementary Figures

## Supplementary figures


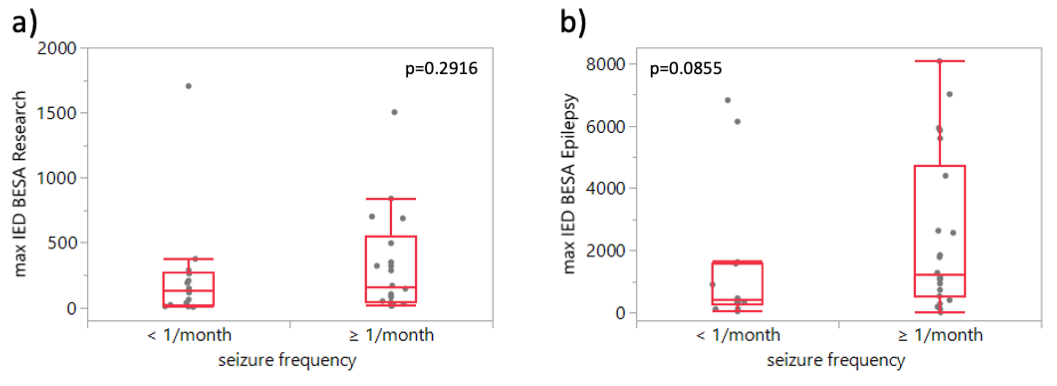


Suppl. Figure 1. IED frequency detected with BESA® Research a) and Epilepsy b) compared according to seizure frequency as recorded by clinical history on admission. No significant difference between the two groups (<1 per month and ≥ 1 per month), and for all groups (daily, weekly, monthly, yearly, rare; data not shown, p=0.4064 and p=0.2224, respectively). N=50, 6 patients excluded from analysis due to unknown seizure frequency, prior to admission.


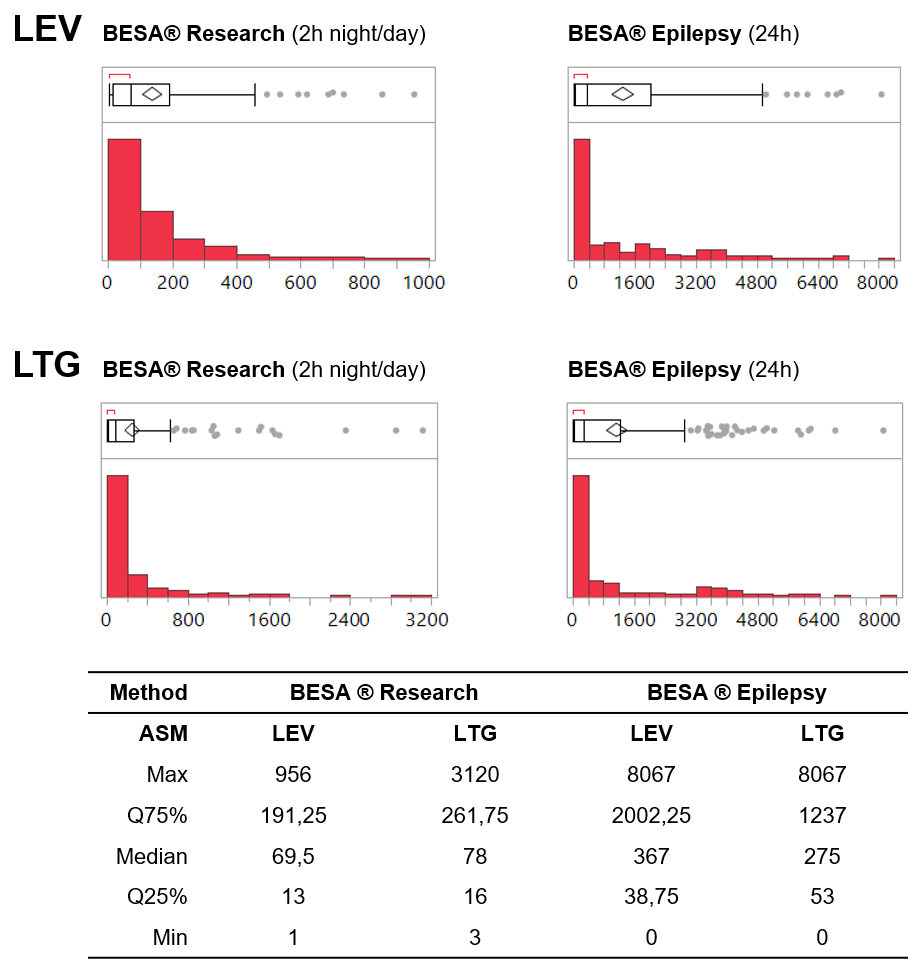


Suppl. figure 2: Absolute IED frequency as determined by both methods, using BESA® Research based on blocks of 2 hours EEG during night and day, respectively, or using BESA® Epilepsy based on 24 hours EEG every day (from 8 AM). Numbers in Max = maximal IED frequency; Q75% = 75% quartile; Median; Q25% = 25% Quartile; Min = minimal IED frequency detected.

**
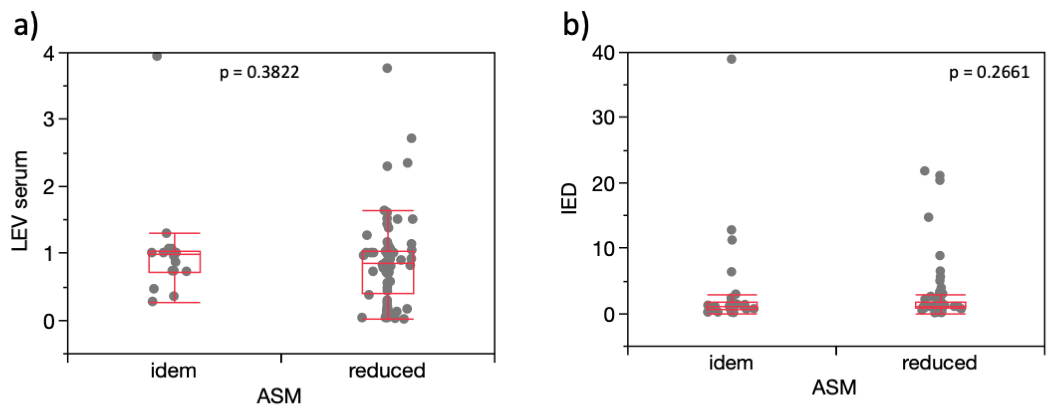
**

Suppl. Figure 3: LEV serum concentration and IED frequency for patients under LEV, who did not reduce the medication (n=5) compared to patients who reduced LEV during the course of the monitoring.

## Supplementary tables

| **Variables** | | **absolute** | **relative** |
| --- | --- | --- | --- |
| **IED frequency** | **BESA Research** | W=0.5310 p<0.0001* | W=0,9417 p<0.0001* |
|  | **BESA Epilepsy** | W=0,6723 p<0.0001* | W=0,8820 p<0.0001* |
| **Serum concentration** | **LEV** | W=0,7110 p<0.0001* | W=0.7975 p<0.0001* |
|  | **LTG** | W=0.9400 p<0.0001* | W=0.9261 p<0.0001* |

Suppl. table 1: Normality test for absolute and relative measures (IED frequency determined using BESA® Research and Epilepsy, and ASM serum concentration for LEV and LTG). Normal distribution has been tested using the Shapiro-Wilk W test (N0=samples drawn from normal distribution), significant p-values indicate non-normal distribution (true for all measures).
